# Effectiveness of International Travel Controls for Delaying Local Outbreaks of COVID-19 

**DOI:** 10.3201/eid2801.211944

**Published:** 2022-01

**Authors:** Bingyi Yang, Sheena G. Sullivan, Zhanwei Du, Tim K. Tsang, Benjamin J. Cowling

**Affiliations:** The University of Hong Kong, Hong Kong, China (B. Yang, Zhanwei Du, T.K. Tsang, B.J. Cowling);; University of Melbourne Peter Doherty Institute for Infection and Immunity, Melbourne, Victoria, Australia (S.G. Sullivan);; Laboratory of Data Discovery for Health Limited, Hong Kong (Z. Du, B.J. Cowling)

**Keywords:** coronavirus disease, 2019 novel coronavirus disease, COVID-19, severe acute respiratory syndrome coronavirus 2, SARS-CoV-2, viruses, respiratory infections, zoonoses, border control, epidemiology

## Abstract

During the coronavirus disease pandemic, international travel controls have been widely adopted. To determine the effectiveness of these measures, we analyzed data from 165 countries and found that early implementation of international travel controls led to a mean delay of 5 weeks in the first epidemic peak of cases.

International travel control (e.g., screening of inbound travelers, requiring quarantines, and even closing borders) has been a key strategy implemented by many countries to limit importations of severe acute respiratory syndrome coronavirus 2 (SARS-CoV-2). However, early in the coronavirus disease (COVID-19) pandemic, the World Health Organization (WHO) did not recommend restricting travel (*1*), and travel controls have not been widely used in previous pandemics (e.g., the 2009–10 influenza pandemic) (*2*,*3*). Limiting international movement has enormous social and economic costs, and the benefits of this strategy (i.e., delaying or averting an epidemic) lack real-world evidence. Previous studies, most of which were simulation studies, suggest that travel restrictions can delay but not prevent local epidemics (*2*–*4*).

To examine the association between implementation of international travel controls and local outbreak progress of COVID-19, we used publicly available data (*5*–*7*; T. Wu et al., unpub. data, https://www.medrxiv.org/content/10.1101/2020.02.25.20027433v1) for January 1–July 31, 2020. Only 14 (8.5%) of the 165 countries studied enacted international travel controls coincident with the lockdown in Wuhan, China (January 23); all controls involved screening inbound travelers (Figure). Enactment of international travel controls peaked ≈3 weeks after WHO declared the pandemic (March 11, 2020), by which time 112 (67.8%) countries completely closed their borders, 44 (26.6%) banned travelers from high-risk regions, and 4 (2.4%) required quarantine for travelers from high-risk regions (Figure; Appendix Figure 1). Of the 165 countries, 90 (54.5%) had imposed at least some restriction before reporting their first COVID-19 case, and 20 (12%) had imposed their strictest restrictions before reporting their first case (Figure; Appendix Figures 1–3). 

**Figure:**
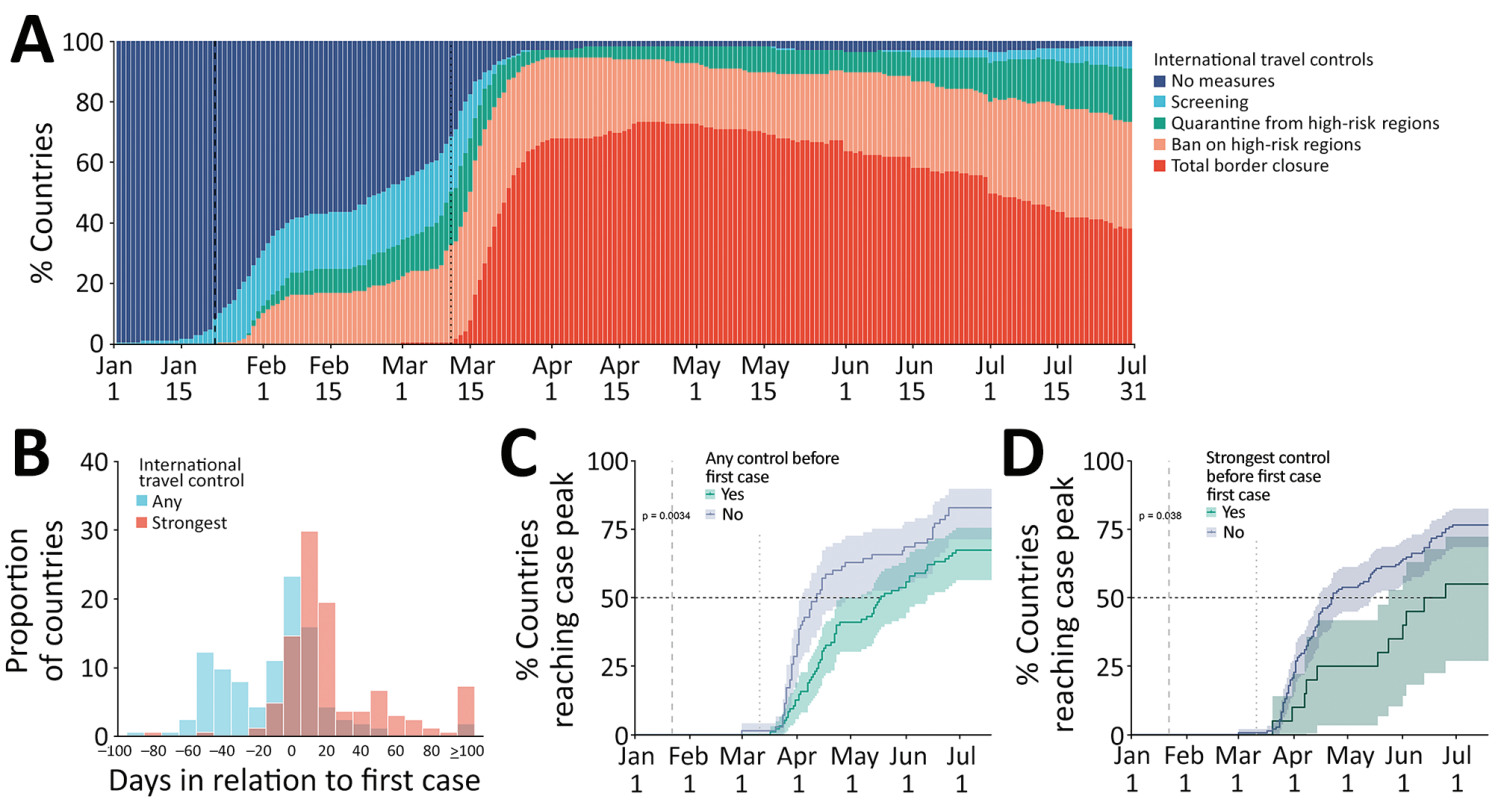
Association between international travel controls and local coronavirus disease (COVID-19) outbreaks in 165 countries, January 1–July 31, 2020. A) Temporal distribution of the international travel controls enacted by the studied countries. Data from (*7*). B) Distribution of the time between a country’s first COVID-19 case and its enactment of any or of the strongest international travel controls. C, D) Probability of reaching first local peak of COVID-19 cases by the time of implementing any (C) or the strongest (D) international travel controls, estimated by using the Kaplan-Meier survival function. Vertical dashed lines in panels B, C, and D indicate the date that Wuhan, China, underwent lockdown; vertical dotted lines indicate the date that the pandemic was declared.

We determined the progress of outbreaks in each country to be the time from January 1, 2020, to the first epidemic peak, which was identified from the modal daily case counts within any 53-day sliding window (i.e., a quarter of the length of the study period) and needed to comprise >10% of the cumulative incidence during the study period (Appendix Figure 2). By July 31, 2020, the first epidemic peak had been reached in 122 (74%) of the studied countries (Appendix Figure 4). In countries that had enacted any international travel controls before their first COVID-19 case, the first peak was reached an average of 36 days (95% CI 10–61 days) later than it was in countries that did not enact controls until after their first case was reported (p<0.01 by log-rank test; Figure). Countries that implemented their strictest international travel controls before detecting any COVID-19 cases reported their first case a median of 57 days (95% CI 14–70 days) later than countries that imposed their strongest controls after the first case was reported (p = 0.04 by log-rank test; Figure).

After adjusting for population density and implementing nonpharmaceutical interventions by using the accelerated failure time model (Appendix), we estimated that the average time to detection of the first case occurred 1.22 (95% CI 1.06–1.41) times later in countries that implemented any restrictions than in countries that implemented no travel restrictions. This time ratio was extended to 1.31 (95% CI 1.02–1.68) if countries implemented their strongest travel restrictions (Table). Such associations still held when adjusting for time-varying nonpharmaceutical interventions by using the Cox model.

**Table:** Estimated time ratios and hazard ratios for comparing selected outcomes in countries that did and did not implement international controls before identifying their first cases of COVID-19, January–July 2020*

Endpoint	Adjusted time ratio (95% CI)†			Adjusted hazard ratio (95% CI)‡	
	Any international controls	The strongest international controls		Any international controls	The strongest international controls
Case peak	1.22 (1.06–1.41)	1.31 (1.02–1.68)		0.66 (0.46–0.93)	0.65 (0.39–1.08)
Death peak	1.23 (1.01–1.51)	0.98 (0.71–1.37)		0.74 (0.53–1.04)	0.90 (0.53–1.55)
Cumulative incidence, no. cases/10,000 population					
0.2	1.20 (1.10–1.31)	1.23 (1.05–1.44)		0.55 (0.38–0.78)	0.61 (0.35–1.04)
1.0	1.26 (1.13–1.42)	1.27 (1.04–1.55)		0.49 (0.35–0.71)	0.90 (0.53–1.51)
5.0	1.25 (1.05–1.49)	1.34 (0.99–1.82)		0.59 (0.41–0.85)	0.90 (0.54–1.51)

*AFT, accelerated failure time; COVID-19, coronavirus disease. †Estimates were obtained from accelerated failure time models with log-logistic distribution, adjusted for population density and the strictest level of each nonpharmaceutical intervention used during the study period for each country. The 2 columns show time ratio of implementing international controls before the country’s first COVID-19 case to that after the country’s first case. ‡Estimates were obtained from Cox proportional hazard models, which adjusted for population density and time-varying nonpharmaceutical interventions during the study period for each country. The 2 columns show hazard ratio of implementing international controls before the country’s first COVID-19 case to that after the country’s first case.

To confirm that these observations were maintained according to alternative measures of epidemic activity, we used the following as outcomes in the models: the time by which COVID-19 deaths first peaked, and attainment of a cumulative incidence of 0.2, 1.0, or 5.0 cases/10,000 persons (by which time peaks had been reached in ≈10%, 30%, and 60% of the countries; Appendix Figure 5). These outcomes may better indicate community spread in countries in which most cases were imported and identified during quarantine (e.g., Fiji), information that was not available from public data. Moreover, outcomes may be better when the epidemic was multimodal (e.g., Guyana) or the country did not experience its main epidemic until later in the study period (e.g., Argentina) (Appendix Figure 2). Both accelerated failure time and Cox models supported earlier observations that enactment of any international travel controls delayed the time in which cumulative incidence rates or deaths peaked. However, enactment of the strongest control was not associated with a reduced time to peak death or cumulative incidence of 5 cases/100,000 persons (Table).

Our work may be influenced by other unmeasured confounders, such as the stringency of international travel controls. We repeated our analyses by removing countries in Asia, in which implementation tended to be more strict, and found that our earlier observations largely held (Appendix Table). In addition, we examined the broader association between international travel controls and local epidemic progression, but we did not examine the roles of specific measures (e.g., quarantine and risk-dependent triage management).

Our findings suggest that implementing international travel controls earlier delayed the initial epidemic peak by ≈5 weeks. Although travel restrictions did not prevent the virus from entering most countries, delaying its introduction bought valuable time for local health systems and governments to prepare to respond to local transmission.

Appendix Supplemental methods and results from study of effectiveness of international travel controls for delaying local outbreaks of coronavirus disease.
