# Supplementary material for: Effectiveness of International Travel Controls for Delaying Local Outbreaks of COVID-19
Source: Emerg Infect Dis. 2022 Jan;28(1):251–3. doi: 10.3201/eid2801.211944 (PMC8714230; doi:10.3201/eid2801.211944)
Supplement: Appendix — Supplemental methods and results from study of effectiveness of international travel controls for delaying local outbreaks of coronavirus disease. [file 21-1944-Techapp-s1.pdf]

Article DOI: https://doi.org/10.3201/eid2801.211944

Effectiveness of International Travel Controls for Delaying Local Outbreaks of COVID-19

Appendix

Data sources

Data on the country-specific time series of reported COVID-19 cases and deaths were obtained from Johns Hopkins Coronavirus Resource Center between Jan 22 to Jul 31, 2020 (*1*). As some countries reported their first COVID-19 case before Jan 22, 2020 (e.g., Thailand and Japan), we obtained the date of first reported COVID-19 case for those countries from T. Wu et al. (unpub. data, https://www.medrxiv.org/content/10.1101/2020.02.25.20027433v1). We only used data before Jul 31, 2020, as many countries started to experience their second wave during or after that time, to which controls targeting local communities would contribute more than that targeting international travels.

We obtained time series of non-pharmaceutical interventions (NPIs) that were implemented by countries from a publicly available database (*2*,*3*), which included international travel restrictions, testing, contact tracing, facial covering, restrictions internal movements, cancel public events, restriction gatherings, close public transport, school closures, stay home requirements and workplace closures. International travel restrictions were classified into five categories with increasing stringency, i.e., no measures, screening, quarantine from high-risk regions, ban on high-risk regions and total border controls (*2*). We characterized the time of any international travel controls as the first date when any international travel controls other than no measures was implemented. We characterized the time of the strongest international travel controls as the first date when the strictest international travel controls during the study period was implemented.

We included countries where have data available for both time series of reported cases and NPIs. We excluded China where the first COVID-19 case was detected.

Methods

We examined the associations between the time of implementing international travel controls and the local outbreak progress of COVID-19 across the studies countries.

Exposure. Binary variable was modelled for the exposure to measure if the international travel control was implemented before or after the country reported its first COVID-19 case. We looked at the time for implementing both any or the strongest international travel control.

Endpoint event. To characterize the local COVID-19 outbreaks progression, we first fit cubic smooth spline to the time series of each country, to avoid the impact of short-term noise caused by reporting. We then used the following five endpoint measurements to characterize the local outbreak progress (Appendix Figure 4):

1) *the first epidemic peak for confirmed COVID-19 cases*, which was the primary outcome used in the main analysis and was defined as the first appeared maxima of cases within any 53-days sliding window (i.e., a quarter of the length of the study period). We also excluded peak with value less than 10% of the cumulative incidence during the study period, to avoid false identification due to sparse cases reported in early phases in some countries. Countries could have multiple peaks during our study period (e.g., United States) and we recorded the first appeared peak. By 31 July 2020, 74% (n = 122) of the countries had experienced their first peak of COVID-19 cases (Appendix Figure 4A).

2) *the first epidemic peak for confirmed COVID-19 death*, which was defined as the same to that of the confirmed cases but using time series of confirmed COVID-19 related deaths. By 31 July 2020, 59% (n = 97) of the countries had experienced their first peak of COVID-19 cases (Appendix Figure 4B).

3) *the cumulative incidence reached 0.2 case per 10,000 persons*, by which 13% of the studied countries had peaked. By 31 July 2020, 87% (n = 143) of the countries had experienced their first peak of COVID-19 cases (Appendix Figure 4C).

4) *the cumulative incidence reached 1 case per 10,000 persons*, by which 30% of the studied countries had peaked. By 31 July 2020, 87% (n = 143) of the countries had experienced their first peak of COVID-19 cases (Appendix Figure 4D).

5) *the cumulative incidence reached 5 cases per 10,000 persons*, by which 57% of the studied countries had peaked. By 31 July 2020, 62% (n = 102) of the countries had experienced their first peak of COVID-19 cases (Appendix Figure 4E).

We noted that the first epidemic peak of COVID-19 cases that we used for our main analysis have many caveats (Appendix Figure 6). For example, some countries (e.g., Fiji) may find most of their cases in quarantine facilities, and therefore the reported cases in these countries may not to be the locally transmitted cases, which could result in misclassification of the first epidemic peak. For some countries had many fluctuations over the study period (e.g., Guyana) or experienced a much larger outbreak during the later of our study period (e.g., Argentina), which could result in right-censoring for the first epidemic peak (i.e., no epidemic peak was observed according to the peak measurement). To validate if our results would be greatly affected by these issues, we used the reaching a certain threshold for cumulative incidence. We believe using the alternative outcome measurements could overcome the abovementioned misclassifications or right-censoring issues, although for some countries with low COVID-19 circulation it could introduce right-censoring as well (e.g., Vietnam, Appendix Figure 6). Nevertheless, results suggested that these measurements were less likely to affect our main conclusion (Table in the main text).

Time-to-event. We calculate the time-to-event as the time between January 1 2020 (the day after the Wuhan cluster was first reported) and the time when the country reached the abovementioned outcome.

Statistical analyses

We plotted the Kaplan–Meier survival curve for the above-mentioned measurements for local COVID-19 outbreaks as endpoint and stratified by whether the country implemented their international travel controls before reporting their first case (Figure panels C, D in the main text and Appendix Figure 4).

We fitted accelerated failure time (AFT) model (Table in the main text) to examine the time ratio of countries which implemented their international travel controls before or after their first case. We adjusted for the country's population density and the strictest level of each NPI that was implemented by the country during the study period. Other NPIs include testing, contact tracing, facial covering, restrictions internal movements, cancel public events, restriction

gatherings, close public transport, school closures, stay home requirements and workplace closures (*2*). We fitted the AFT with four distributions, i.e., exponential, Weibull, lognormal and loglogistic. We presented the results from the AFT model with loglogistic distribution as the Akaike information criterion (AIC) suggested it provided the best model fit.

Country adjusted their NPIs along with the progress of local COVID-19 outbreak. So, we also fitted a Cox proportional hazard model (Appendix Table), to allow for modelling individual NPIs as time-varying variables. We reported the hazard ratio of reaching the outcome measurements between countries that implemented their international travel controls before or after their first case, after adjusting for population density and time-varying NPIs.

There may be other unmeasured confounders that could lead the observed associations between earlier enactment of international travel controls and delayed local epidemic progressions. For instance, countries where implemented travel controls before their first COVID-19 case may also be more precautious and adherent when implanting other non-pharmaceutical interventions. We performed a sensitivity analysis by fitting the AFT and Cox models with data that excluding Asian countries, where tended to have stricter enactment, higher adherence and more precautious when implementing these control measures (Appendix Table). In total 42 Asian countries were excluded in the sensitivity analyses, which are Afghanistan, Azerbaijan, Bahrain, Bangladesh, Bhutan, Cambodia, Cyprus, Georgia, India, Indonesia, Iran, Iraq, Israel, Japan, Jordan, Kazakhstan, Kuwait, Kyrgyzstan, Laos, Lebanon, Malaysia, Mongolia, Myanmar, Nepal, Oman, Pakistan, Philippines, Qatar, Saudi Arabia, Singapore, South Korea, Sri Lanka, Syria, Tajikistan, Thailand, Timor-Leste, Turkey, United Arab Emirates, Uzbekistan, Vietnam and Yemen.

References

1. Dong E, Du H, Gardner L. An interactive web-based dashboard to track COVID-19 in real time. Lancet Infect Dis. 2020;20:533–4. PubMed https://doi.org/10.1016/S1473-3099(20)30120-1

2. Policy Responses to the Coronavirus Pandemic - Statistics and Research - Our World in Data [cited 2021 Aug 2]. https://ourworldindata.org/policy-responses-covid

3. Hale T, Angrist N, Goldszmidt R, Kira B, Petherick A, Phillips T, et al. A global panel database of pandemic policies (Oxford COVID-19 Government Response Tracker). Nat Hum Behav. [cited 2021 Aug 2]. https://www.nature.com/articles/s41562-021-01079-8

Appendix Table. Estimated time ratios and hazard ratios for selected outcomes comparing countries which implemented international controls before identification of their first cases versus those that did not, after removing Asian countries*

	Adjusted time ratio (from AFT model)†		Adjusted hazard ratio (from Cox model)‡	
Endpoint	Any international controls	The strongest international controls	Any international controls	The strongest international controls
Case peak	1.27 (1.08, 1.51)	1.31 (0.95, 1.79)	0.67 (0.44, 1.02)	0.70 (0.37, 1.30)
Death peak	1.30 (1.05, 1.60)	1.01 (0.72, 1.42)	0.71 (0.47, 1.07)	0.93 (0.49, 1.78)
Cumulative incidence				
0.2 per 10,000	1.25 (1.14, 1.37)	1.34 (1.14, 1.58)	0.42 (0.27, 0.67)	0.42 (0.20, 0.88)
1 per 10,000	1.35 (1.20, 1.53)	1.40 (1.13, 1.74)	0.34 (0.22, 0.53)	0.79 (0.42, 1.48)
5 per 10,000	1.43 (1.17, 1.76)	1.69 (1.17, 2.44)	0.45 (0.29, 0.69)	0.73 (0.40, 1.35)

*A total of 42 Asian countries were excluded in the analyses.

†Estimates were obtained from accelerated failure time (AFT) models with loglogistic distribution, adjusted for population density and the strictest level of each NPI used during the study period for each country. The two columns show time ratio of implementing international controls before the country's first COVID-19 case to that after the country's first case.

‡Estimates were obtained from Cox proportional hazard models, which adjusted for population density and time-varying NPIs during the study period for each country. The table shows hazard ratio of implementing international controls before the country's first COVID-19 case to that after the country's first case.

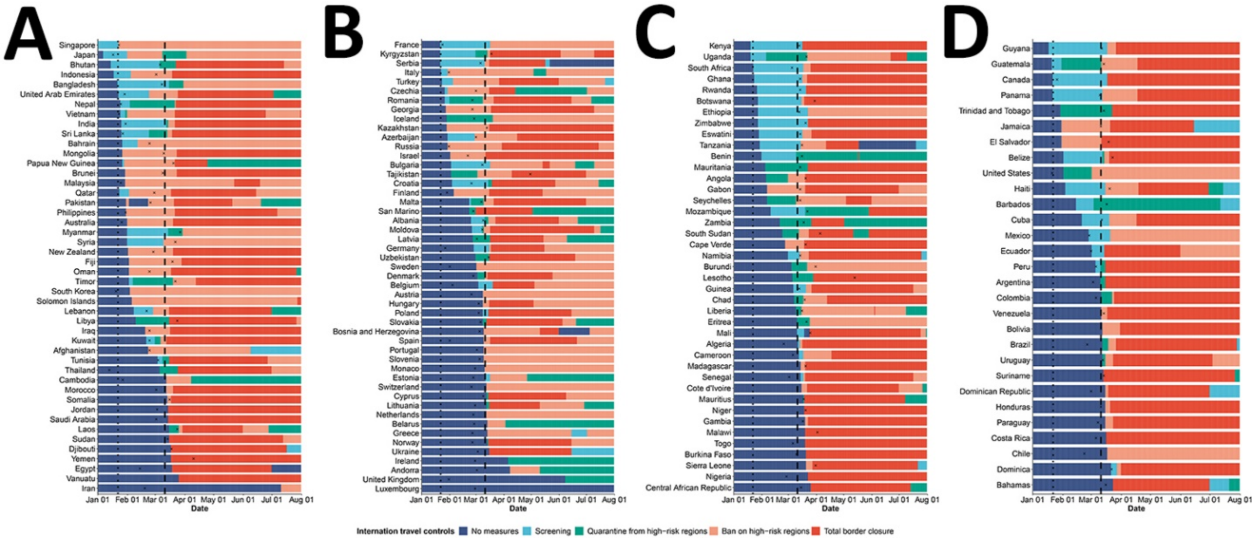

Appendix Figure 1. Temporal distribution of international travel control implementation in 165 countries, 1 January to 31 July 2020. Data were derived from https://ourworldindata.org/policy-responses-covid. Cross indicates the time when country reported its first case.

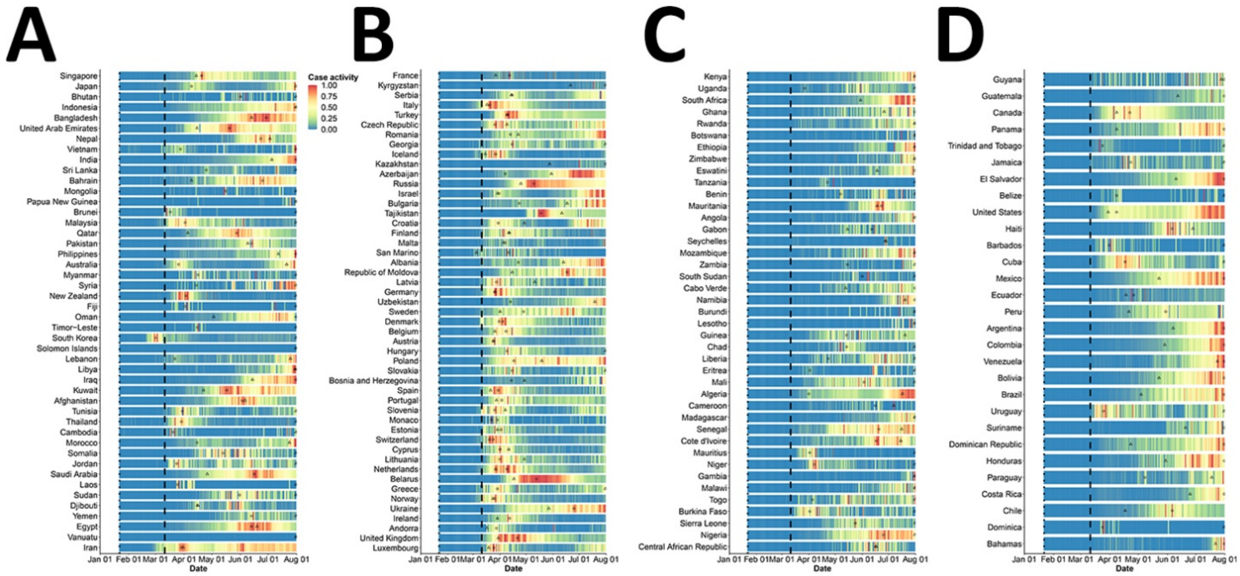

Appendix Figure 2. Temporal distribution of confirmed COVID-19 cases in 165 countries, 1 January to 31 July 2020. Data were derived from (*1*). Circle and triangle indicate the time when the country reached its first epidemic peak of cases and 5 cases per 10,000, respectively.

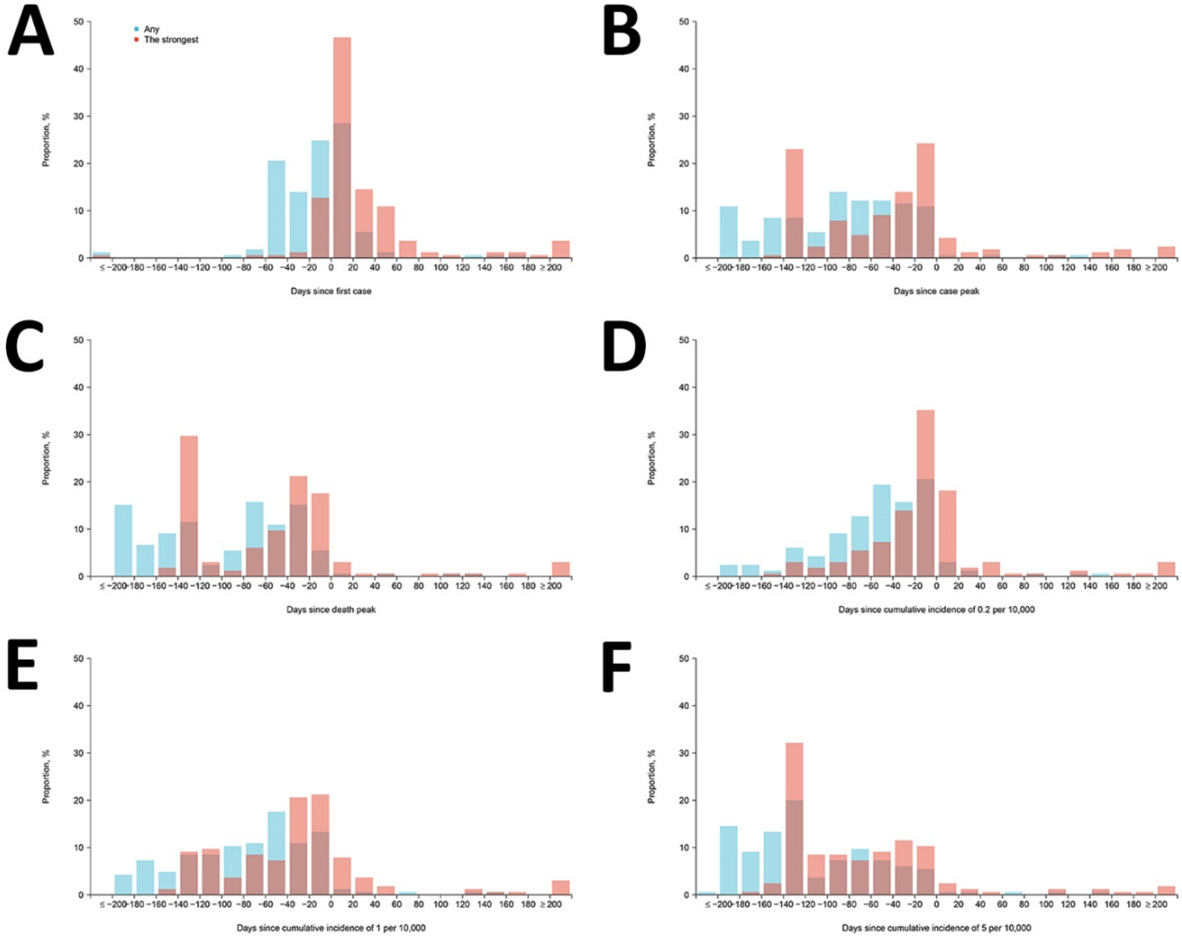

Appendix Figure 3. Distribution of times from enactment of international travel controls to local COVID-19 epidemic progression, as measured by the time of the first reported case (A), first epidemic peak of cases (B), first epidemic peak of deaths (C), reached 0.2 case per 10,000 (D), reached 1 case per 10,000 (E) and reached 5 cases per 10,000 (F).

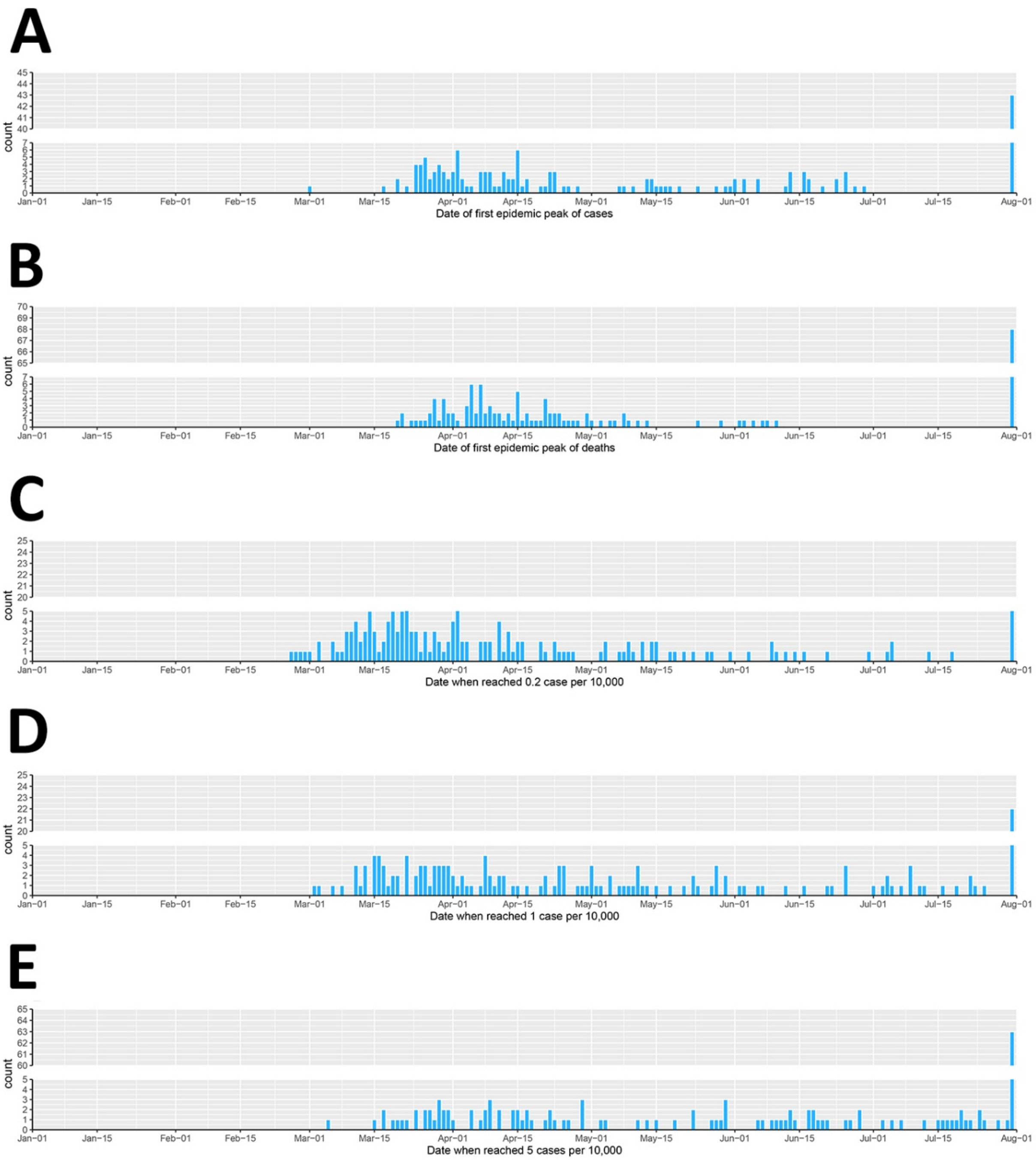

Appendix Figure 4. COVID-19 epidemic milestones for 165 countries: first epidemic peak of cases (A); first epidemic peak of deaths (B); cumulative incidence reached 0.2 COVID-19 case per 10,000 persons (C); cumulative incidence reached 1 COVID-19 case per 10,000 persons (D); and cumulative incidence reached 5 COVID-19 cases per 10,000 persons (E). Count of countries is shown on the y-axis.

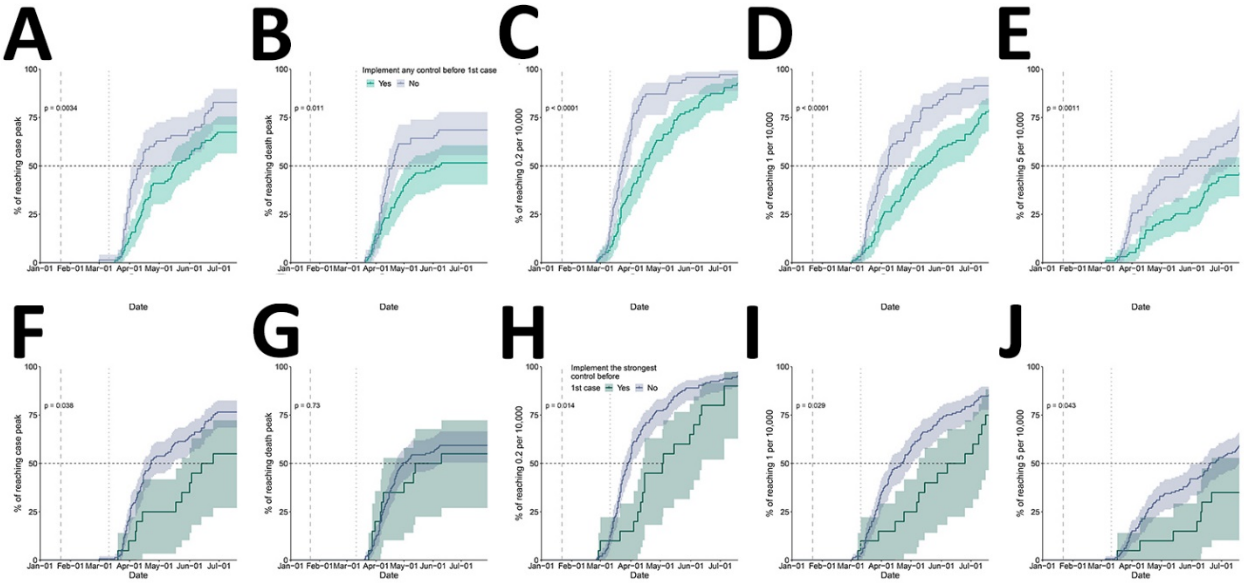

Appendix Figure 5. Association between international travel restrictions and local COVID-19 outbreaks in 165 countries, 1 January to 31 July, 2020. Countries were stratified by the start time of any (A-E) or the strongest (F-J) international travel controls. Columns from left to right were results from analyses that used endpoint of the peak of COVID-19 cases (A, F), the peak of COVID-19 deaths (B, G), reaching cumulative incidence of 0.2 per 10,000 (C, H), reaching cumulative incidence of 1 per 10,000 (D, I) and reaching cumulative incidence of 5 per 10,000 (E, J). Vertical dashed and dotted lines represent the time of Wuhan lockdown and the declaration of pandemic, respectively.

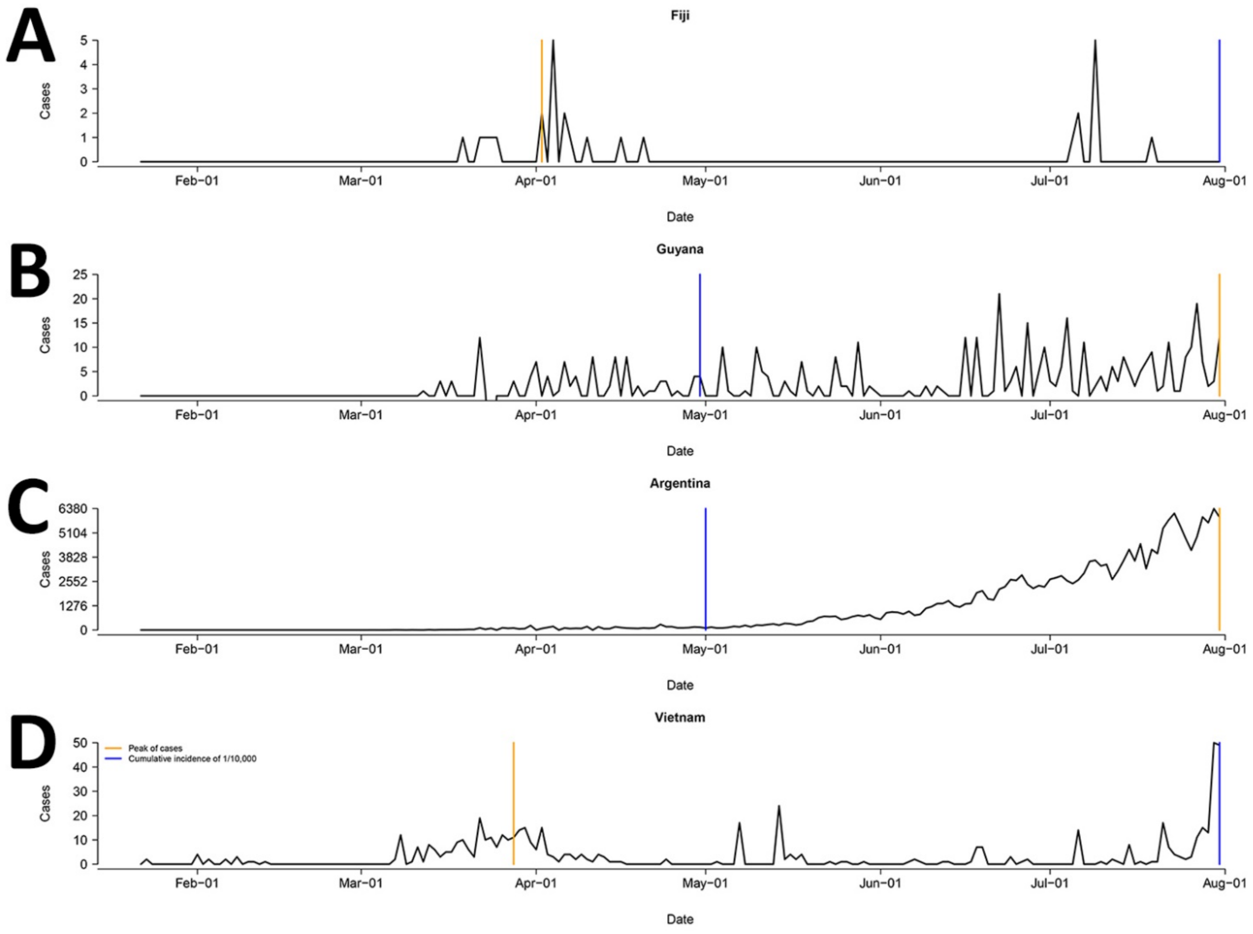

Appendix Figure 6. Representative countries for measuring local COVID-19 outbreak progression.
